# Evolving Radiological Approaches in the Diagnosis and Monitoring of Arachnoiditis Ossificans

**DOI:** 10.7759/cureus.68399

**Published:** 2024-09-01

**Authors:** Sumerjit Singh, Ripudaman Singh, Shivansh Luthra, Abhinandan Singla, FNU Tanvir, Harman Antaal, Agamjit Singh, Harmanjot Singh, Jaskaran Singh, Meet Sirjana Kaur

**Affiliations:** 1 Diagnostic Radiology, Government Medical College Amritsar, Amritsar, IND; 2 Internal Medicine, Government Medical College Amritsar, Amritsar, IND; 3 Medicine, Government Medical College Amritsar, Amritsar, IND; 4 Internal Medicine, Government Medical College Patiala, Patiala, IND; 5 Psychiatry, Punjab Institute of Medical Sciences, Jalandhar, IND; 6 Internal Medicine, The White Medical College and Hospital, Bungal, IND; 7 Internal Medicine, Sri Guru Ram Das University of Health Sciences and Research, Amritsar, IND

**Keywords:** multimodal imaging approach, artificial intelligence, advanced imaging techniques, disease monitoring, spinal imaging, calcification, magnetic resonance imaging, computed tomography, radiological diagnosis, arachnoiditis ossificans

## Abstract

Arachnoiditis ossificans (AO) is a rare and complex neurological condition characterized by pathological calcification or ossification of the arachnoid membrane. Arachnoiditis ranks as the third most frequent cause of failed back surgery syndrome (FBSS). This narrative review explores the evolving radiological approaches in its diagnosis and monitoring. The historical perspective traces the progression from plain radiographs to advanced imaging techniques. Current radiological modalities, including X-ray, computed tomography (CT), and magnetic resonance imaging (MRI), are discussed, highlighting their respective roles, advantages, and limitations. Emerging and advanced imaging modalities, such as high-resolution CT, 3T and 7T MRI, and PET/CT or PET/MRI, are examined for their potential to enhance diagnostic accuracy and monitoring capabilities. A comparative analysis of these imaging modalities considers their sensitivity, specificity, cost-effectiveness, and radiation exposure implications. The review also explores the crucial role of imaging in disease monitoring and treatment planning, including follow-up protocols, evaluation of disease progression, and guidance for interventional procedures. Future directions in the field are discussed, focusing on promising research areas, the potential of artificial intelligence and machine learning in image analysis, and identified gaps in current knowledge. The review emphasizes the importance of a multimodal imaging approach and the need for standardized protocols. It concludes that while significant advancements have been made, further research is necessary to fully understand the correlation between imaging findings and clinical outcomes. The continued evolution of radiological approaches is expected to significantly improve patient care and outcomes in AO.

## Introduction and background

Arachnoiditis ossificans (AO) is a rare and complex condition characterized by the pathological calcification or ossification of the arachnoid mater, one of the protective membranes surrounding the brain and spinal cord [1]. This condition can lead to severe neurological deficits, chronic pain, and significant morbidity, making accurate diagnosis and monitoring crucial for patient management [2].

The etiology of AO remains poorly understood, with proposed causes including trauma, infection, inflammation, and iatrogenic factors such as intrathecal contrast media or steroid injections [3]. The insidious onset and non-specific symptoms often result in delayed diagnosis, highlighting the importance of advanced radiological techniques in identifying and characterizing this condition [4].

Radiological approaches play a pivotal role in the diagnosis and monitoring of AO. Traditional imaging modalities such as X-rays and computed tomography (CT) have been instrumental in detecting calcifications, while magnetic resonance imaging (MRI) has revolutionized our ability to visualize soft tissue changes and assess the extent of arachnoid involvement [5]. As technology advances, newer imaging techniques offer the potential for earlier detection, more precise characterization of lesions, and improved monitoring of disease progression [6].

The aim of this narrative review is to provide a comprehensive overview of the evolving radiological approaches in the diagnosis and monitoring of AO. We explored the historical context of imaging in this condition, evaluated current radiological techniques, and discussed emerging modalities that show promise in enhancing diagnostic accuracy and treatment planning. Additionally, this review assessed the comparative effectiveness of various imaging methods, considering their clinical implications, and identifying areas for future research [7]. By synthesizing the latest evidence and expert opinions, we seek to offer clinicians and radiologists a robust framework for approaching the radiological evaluation of AO. This review aims to contribute to improved patient outcomes through earlier diagnosis, more effective monitoring, and better-informed treatment decisions in this challenging neurological condition [8].

AO is characterized by the abnormal deposition of calcium or bone within the arachnoid membrane of the central nervous system. The pathophysiology involves a complex interplay of inflammatory processes, fibrosis, and dysregulated calcium metabolism [9]. The etiology of the inflammation has three main causes. Trauma surgery, specifically complications after multiple back surgeries, can result in blood penetration in the subarachnoid space, leading to inflammation. Chemical exposure is another cause, which includes contact with oil-based radiographic contrast agents used in myelograms or drugs used for epidural injections. The third cause is infection, where viral or bacterial meningitis, tuberculosis, and syphilis can affect the spine and trigger inflammation [9]. Initially, an insult to the arachnoid membrane triggers an inflammatory response, leading to the proliferation of arachnoid cells and fibroblasts. Over time, this chronic inflammation results in the formation of dense fibrous tissue, which can subsequently calcify or ossify [10]. There are notable distinctions in the presentation of arachnoiditis depending on its origin. When resulting from surgical procedures, the inflammation tends to be confined to a specific area. In contrast, arachnoiditis stemming from epidural injections typically manifests in a more widespread manner, affecting a broader region of the arachnoid membrane.

The clinical presentation of AO is often nonspecific and can mimic other neurological conditions. Patients typically experience chronic back pain, radiculopathy, and sensory disturbances [11]. As the disease progresses, more severe symptoms may develop, including motor deficits, bowel and bladder dysfunction, and in extreme cases, paralysis [12]. The onset is usually insidious, with symptoms gradually worsening over months to years.

Diagnosing and monitoring AO presents several challenges. The rarity of the condition and its nonspecific symptoms often lead to delayed or misdiagnosis [13]. Radiological findings can be subtle in the early stages, requiring high clinical suspicion and expertise in interpreting imaging studies [14]. Additionally, the slow progression of the disease necessitates long-term follow-up, which can be challenging due to the limitations of repeated imaging, especially with modalities involving radiation exposure [15].

Another significant challenge is differentiating AO from other spinal pathologies, such as degenerative disc disease or spinal stenosis, which may present with similar symptoms [16]. This differentiation is crucial for appropriate management and prognosis. Furthermore, the lack of standardized diagnostic criteria and imaging protocols adds to the complexity of accurate diagnosis and effective monitoring of disease progression [17].

## Review

Historical perspective

The radiological diagnosis of AO has evolved significantly since its first description in the medical literature. Early approaches relied heavily on plain radiographs, which could detect advanced calcifications but often missed subtle or early-stage changes [1]. These X-rays were limited in their ability to visualize soft tissue involvement and the extent of arachnoid membrane alterations.

Myelography, introduced in the 1920s, marked a significant advancement in spinal imaging [18]. This technique involved injecting a contrast medium into the subarachnoid space, allowing for better visualization of the spinal cord and nerve roots. However, myelography had its own limitations, including the invasive nature of the procedure and the risk of exacerbating arachnoiditis due to the irritant effects of early contrast agents [19].

The advent of computed tomography (CT) in the 1970s revolutionized the diagnosis of spinal pathologies, including AO [20]. CT scans provided cross-sectional images with superior bone detail, enabling better visualization of calcifications. Nevertheless, CT was limited in its soft tissue resolution and ability to detect early inflammatory changes in the arachnoid membrane.

Traditional imaging techniques faced several limitations in diagnosing and monitoring AO. These included poor soft tissue contrast, the inability to detect early-stage disease, limited visualization of the extent of arachnoid involvement, and, in some cases, exposure to ionizing radiation [5]. Additionally, these methods often struggled to differentiate AO from other spinal pathologies, leading to potential misdiagnosis or delayed treatment [8].

Current radiological techniques

The diagnosis and monitoring of AO rely on a combination of imaging modalities, each offering unique advantages and limitations. This section explores the current radiological techniques used in clinical practice.

X-ray Imaging

Plain radiography remains a valuable initial screening tool due to its wide availability, low cost, and ability to provide a quick overview of bony structures [9]. X-rays are particularly useful in detecting advanced stages of AO, where significant calcification or ossification has occurred. However, this modality has several limitations. It lacks soft tissue resolution, making it ineffective for visualizing early-stage disease or assessing the extent of arachnoid involvement. Additionally, X-rays provide only two-dimensional images, which can lead to difficulties in precise localization of lesions [11].

On X-ray imaging, AO typically appears as linear or plaque-like calcifications along the spinal cord or nerve roots. In advanced cases, these calcifications may form a continuous sheet-like appearance, sometimes described as a "tram-track" sign [3]. However, it's important to note that these findings are often only visible in the later stages of the disease, potentially leading to delayed diagnosis if relied upon exclusively.

Computed Tomography (CT)

CT plays a crucial role in the diagnosis of AO, offering superior bone detail and the ability to detect smaller calcifications compared to plain radiographs [16]. Its multi-planar reconstruction capabilities allow for better localization and assessment of the extent of calcification. CT is particularly useful in evaluating the relationship between calcifications and adjacent bony structures, which is essential for surgical planning if intervention becomes necessary.

**Figure 1:**
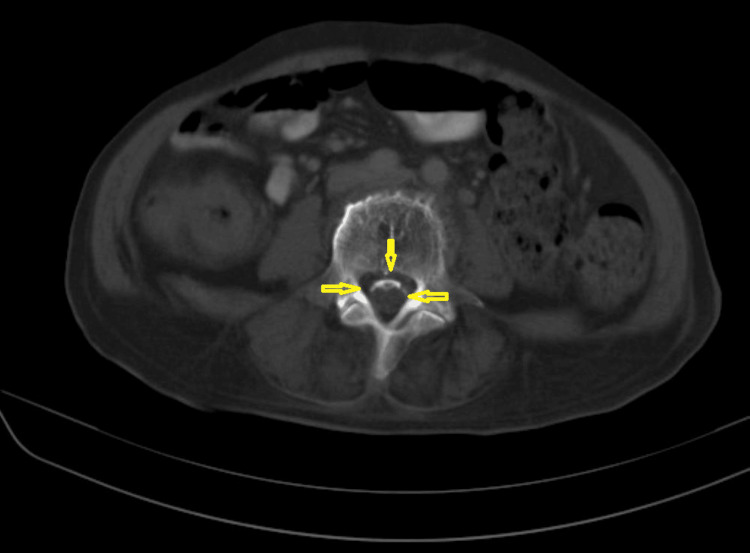
CT is the best investigation tool to visualize the ossified intraspinal lesion and nerve roots. There are curvilinear dural calcifications, intrathecal coarse calcifications, and ossified individual nerve roots adhered to the posterior thecal sac. (Image published under Creative Commons License; image credits: case courtesy of Noriza Zainol Abidin, Radiopaedia.org, rID: 51394)

**Figure 2:**
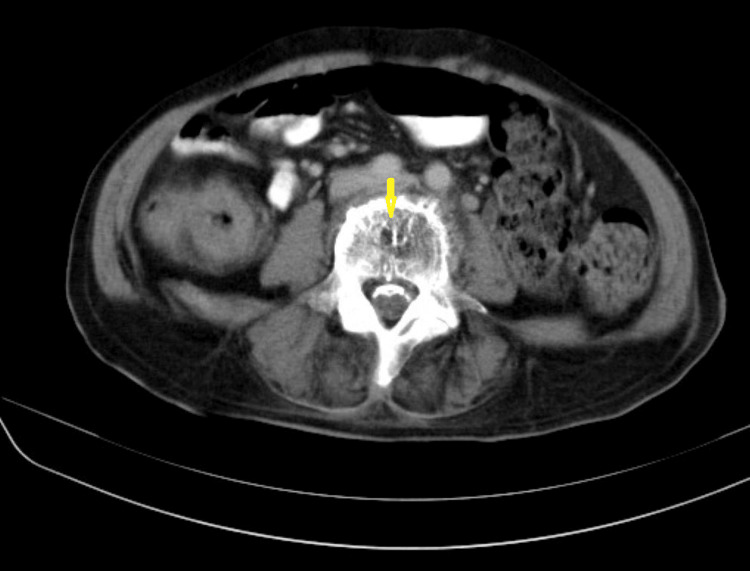
Diffuse, dense curvilinear calcifications are seen along the anterior spinal dura mater, extending from L1 to L5 level. Multiple intrathecal calcifications suggestive of cauda equina ossifications. Some of the intrathecal calcifications follow the course of traversing nerve roots. (Image published under Creative Commons License; image credits: case courtesy of Noriza Zainol Abidin, Radiopaedia.org, rID: 51394)

On CT imaging, AO typically presents as hyperdense linear or nodular calcifications within the spinal canal (Figures 1, 2). These calcifications often conform to the contours of the spinal cord or nerve roots, creating a characteristic "sugar-coating" appearance [14]. In more advanced cases, CT may reveal thickening and calcification of the arachnoid membrane, sometimes forming a complete ring around the spinal cord. Additionally, CT can detect associated findings such as spinal canal stenosis or nerve root compression, which are important for assessing the clinical impact of the condition [12].

Magnetic Resonance Imaging (MRI)

MRI has revolutionized the diagnosis and monitoring of AO due to its superior soft tissue contrast and multiplanar imaging capabilities [5]. Conventional MRI sequences, including T1-weighted, T2-weighted, and short tau inversion recovery (STIR) images, provide valuable information about the extent of arachnoid involvement, spinal cord compression, and associated inflammatory changes.

**Figure 3:**
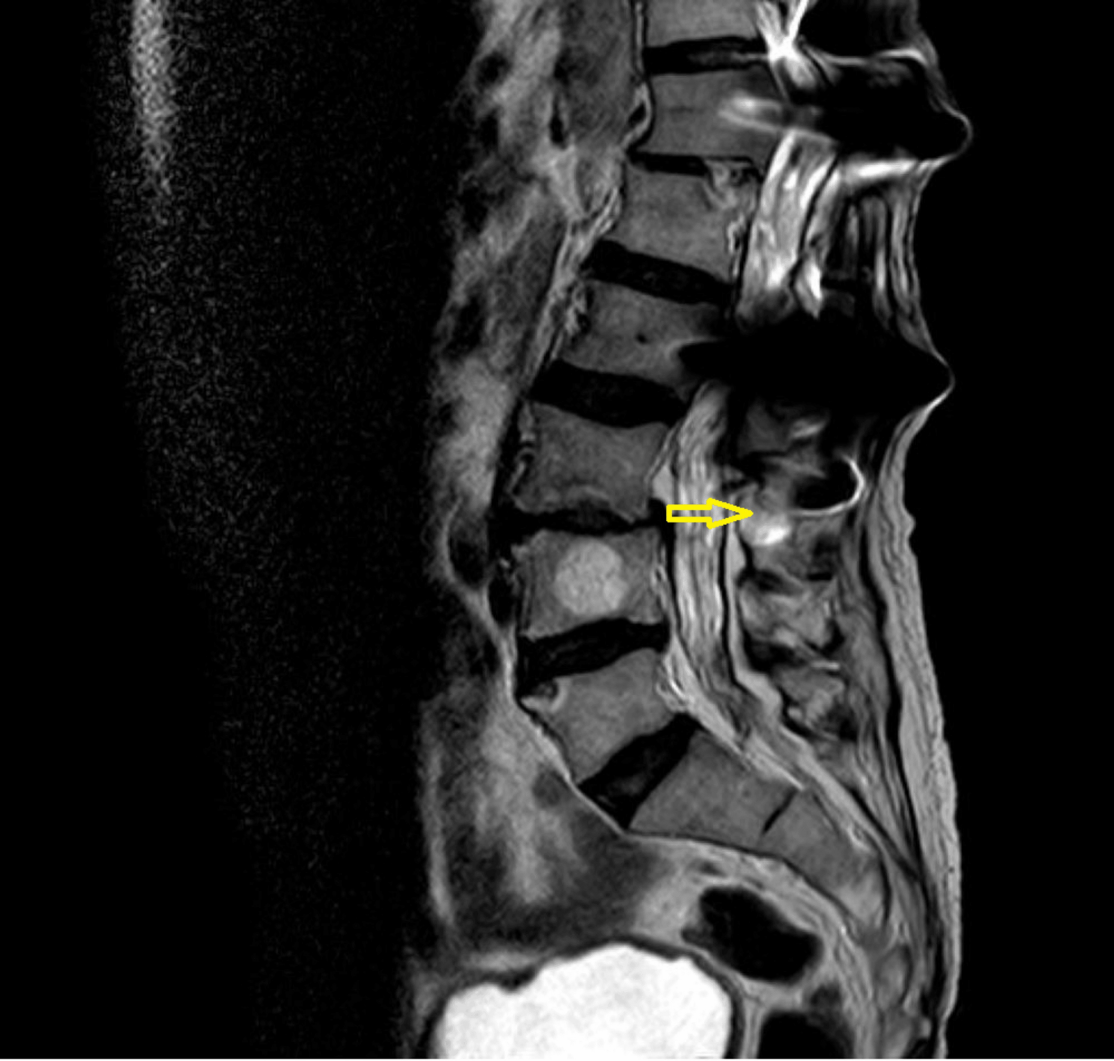
MRI is favorable in depicting arachnoiditis. Coronal T2-weighted image shows diffuse abnormal circumferential thickening of thecal sac with blooming artifact, concordant with dural calcifications seen on CT image. (Image published under Creative Commons License; image credits: case courtesy of Noriza Zainol Abidin, Radiopaedia.org, rID: 51394)

**Figure 4:**
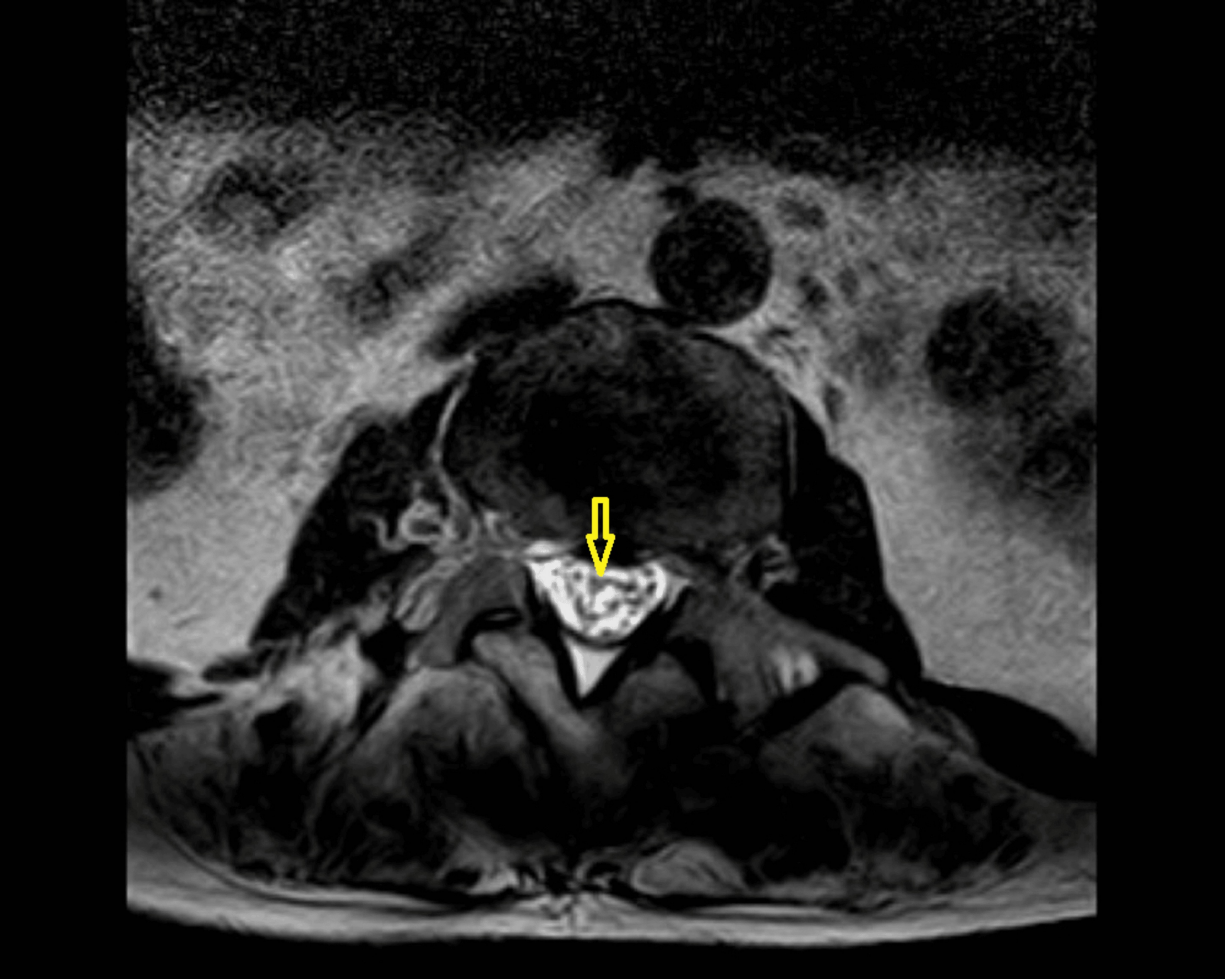
Axial T2-weighted image show displacement of the nerve roots of the cauda equina in the initial images. Abnormal thickening and clumping of the cauda equina with intrathecal hypointense signal abnormality seen at distal lumbar, consistent with sequelae of arachnoiditis. (Image published under Creative Commons License; image credits: case courtesy of Noriza Zainol Abidin, Radiopaedia.org, rID: 51394)

On T1-weighted images, calcifications typically appear as hypointense areas, while on T2-weighted images, they may show variable signal intensity depending on the degree of calcification (Figures 3, 4) [8]. STIR sequences are particularly useful in detecting edema and inflammation associated with active disease. Gadolinium-enhanced T1-weighted images can help identify areas of active inflammation and differentiate between adhesive and non-adhesive arachnoiditis [21].

Recent advancements in MRI technology have introduced several innovative techniques that offer additional insights into the pathophysiology and progression of AO:

Diffusion-weighted imaging (DWI): DWI and its quantitative counterpart, diffusion tensor imaging (DTI), provide information about water molecule movement within tissues. In AO, DWI can help assess the integrity of white matter tracts and detect early microstructural changes in the spinal cord that may not be visible on conventional sequences [22].

Magnetic resonance spectroscopy (MRS): Although more commonly used in brain imaging, MRS has shown potential in spinal cord pathologies. It allows for the non-invasive measurement of metabolites, which could provide insights into the biochemical changes associated with AO [23].

Susceptibility-weighted imaging (SWI): This technique enhances the visualization of calcifications and hemosiderin deposits, potentially improving the detection of small or early calcifications in AO [24].

High-resolution MRI: The use of 3T and 7T MRI scanners offers increased signal-to-noise ratio and spatial resolution, allowing for more detailed visualization of the spinal cord and arachnoid membrane. This can be particularly valuable in detecting subtle changes in early-stage disease [25].

Dynamic contrast-enhanced MRI: This technique allows for the assessment of blood-spinal cord barrier permeability, which may be altered in inflammatory conditions like AO [26].

Each of these radiological techniques contributes uniquely to the diagnosis and monitoring of AO. While X-rays and CTs excel in detecting calcifications, MRI provides superior soft tissue detail and the ability to assess inflammatory changes. The choice of imaging modality often depends on the clinical presentation, stage of the disease, and availability of technology. In many cases, a multimodal approach combining different imaging techniques may offer the most comprehensive evaluation of this complex condition [17].

Emerging and advanced imaging modalities

As our understanding of AO evolves, so do the imaging technologies used to diagnose and monitor this condition. This section explores emerging and advanced imaging modalities that show promise in enhancing our ability to detect, characterize, and track the progression of this rare spinal pathology.

High-resolution CT

Advancements in CT technology have led to the development of high-resolution CT (HRCT) scanners, which offer significant improvements over conventional CT in imaging AO [27]. HRCT demonstrates improved detection of early-stage calcifications, potentially enabling earlier diagnosis by identifying small, developing calcifications before they become apparent on conventional imaging [28]. The higher resolution allows for enhanced characterization of calcification patterns, providing more detailed visualization of the distribution and morphology of calcifications, which aids in differentiating AO from other spinal pathologies [29]. Additionally, HRCT offers a better assessment of surrounding structures, providing clearer images of the relationship between calcifications and adjacent neural structures, which is crucial for surgical planning and assessing potential complications [30]. However, the increased radiation dose associated with this technology remains a concern, particularly for long-term monitoring. This limitation underscores the importance of judicious use and the need for optimized low-dose protocols [31].

3T and 7T MRI

The advent of high-field strength MRI scanners, particularly 3T and 7T systems, has opened new avenues for imaging AO. These advanced MRI systems offer several advantages over conventional 1.5T scanners. They provide an increased signal-to-noise ratio (SNR), allowing for better visualization of fine anatomical details and subtle pathological changes [32]. The enhanced spatial resolution of 3T and 7T MRI can produce images with submillimeter resolution, enabling more precise characterization of arachnoid membrane alterations and small calcifications [33]. Higher field strengths offer improved contrast between different tissue types, potentially aiding in the differentiation between inflammation, fibrosis, and calcification [34]. These systems also facilitate the use of sophisticated MRI sequences such as susceptibility-weighted imaging (SWI), which is particularly sensitive to calcifications and iron deposits [35]. The 7T MRI, while still primarily used in research settings, shows particular promise for imaging AO. Its ultra-high field strength allows for unprecedented spatial resolution and contrast, potentially revealing microstructural changes in the spinal cord and arachnoid membrane that are invisible at lower field strengths [36]. However, the use of high-field MRI is not without challenges. These include increased susceptibility artifacts, especially near tissue-bone interfaces, and potential safety concerns related to the stronger magnetic field. Additionally, the limited availability and high cost of these systems may restrict their widespread use in clinical practice [37].

PET/CT or PET/MRI

Positron emission tomography (PET) combined with CT or MRI represents a cutting-edge approach to imaging AO. While not yet widely used for this specific condition, these hybrid imaging modalities offer unique insights into both the structural and functional aspects of the disease [38]. PET/CT or PET/MRI can provide metabolic information, revealing areas of increased metabolic activity and potentially identifying regions of active inflammation or accelerated calcification [39]. These techniques also allow for the assessment of disease activity, potentially differentiating between active and chronic stages of the disease, which can guide treatment decisions [40]. Additionally, serial PET imaging could be used to evaluate treatment response by monitoring changes in metabolic activity over time [41].

The choice of radiotracer is crucial in PET imaging. While 18F-FDG (fluorodeoxyglucose) is commonly used to detect inflammation, more specific tracers such as 18F-NaF (sodium fluoride) might be particularly useful for imaging calcification processes in AO [42]. The combination of PET with high-resolution CT or MRI allows for precise anatomical localization of metabolic abnormalities, providing a comprehensive assessment of the disease. However, the use of PET in spinal imaging is still evolving, and more research is needed to establish its role in the management of AO [43].

Other Novel Techniques

Several emerging imaging techniques show potential for improving the diagnosis and monitoring of AO. Dual-energy CT (DECT) uses two different X-ray energy levels to provide additional information about tissue composition, enhancing the differentiation between calcifications and other high-density materials and potentially improving the specificity of diagnosis [44]. Ultrashort echo time (UTE) MRI sequences allow for the visualization of tissues with very short T2 relaxation times, such as calcifications, which could improve the detection of early-stage calcifications that may be missed by conventional MRI sequences [45]. Magnetic resonance elastography (MRE), although primarily used for abdominal imaging, has shown promise in assessing tissue stiffness in the central nervous system and could potentially provide information about the mechanical properties of the arachnoid membrane and surrounding tissues in AO [46].

The application of artificial intelligence (AI) and machine learning to imaging data is an exciting frontier in radiology. In the context of AO, AI could potentially assist in early detection, automated quantification of disease progression, and prediction of treatment outcomes [47]. Additionally, the development of targeted molecular probes in molecular imaging could allow for more specific imaging of the biological processes involved in AO, such as inflammation or abnormal calcium metabolism [48]. These emerging techniques offer promising avenues for enhancing our understanding and management of AO.

The integration of these advanced imaging techniques into the diagnostic workup of AO has the potential to revolutionize our approach to this challenging condition. By providing earlier detection, more accurate characterization, and better monitoring of disease progression, these modalities may lead to improved patient outcomes through more timely and targeted interventions.

Comparative analysis of imaging modalities

The diagnosis and monitoring of AO often require a multimodal imaging approach. Each imaging modality offers unique strengths and limitations, making a comparative analysis essential for optimal patient management.

Sensitivity and Specificity

X-ray imaging, while widely available, has limited sensitivity for early-stage AO. It can detect advanced calcifications but lacks specificity, as other conditions may present similar radiographic findings [9].

Computed tomography (CT) offers superior sensitivity for detecting calcifications compared to X-rays. High-resolution CT further improves sensitivity, potentially identifying early-stage disease. However, its specificity may be limited in differentiating between calcifications and other high-density structures [3].

Magnetic resonance imaging (MRI) provides excellent soft tissue contrast, making it highly sensitive for detecting arachnoid membrane changes and associated inflammation. While conventional MRI may have lower sensitivity for calcifications compared to CT, advanced techniques like susceptibility-weighted imaging can improve calcium detection. MRI generally offers higher specificity in characterizing the full spectrum of AO [5].

PET/CT or PET/MRI combines the high sensitivity of PET for metabolic activity with the anatomical detail of CT or MRI. This hybrid approach potentially offers both high sensitivity and specificity, particularly in assessing disease activity and progression [39].

Cost-effectiveness

X-ray imaging is the most cost-effective option for initial screening but may lead to increased overall costs if additional imaging is frequently required due to its limitations [49].

CT is more expensive than X-ray but less costly than MRI. Its ability to detect calcifications effectively may reduce the need for additional imaging in some cases, potentially improving cost-effectiveness [50].

MRI, while the most expensive of these three modalities, provides comprehensive information that may obviate the need for multiple imaging studies. In complex cases, the detailed information provided by MRI may lead to more accurate diagnosis and treatment planning, potentially reducing long-term healthcare costs [51].

PET/CT and PET/MRI are the most expensive options. Their use in AO is still evolving, and their cost-effectiveness remains to be fully established. However, in cases where assessment of disease activity is crucial, these modalities may provide valuable information that justifies their higher cost [44].

Radiation Exposure Considerations

X-ray imaging involves relatively low radiation exposure, but repeated examinations for monitoring can lead to cumulative radiation effects [52]. CT, on the other hand, delivers higher radiation doses compared to X-rays, which is a significant concern, especially for young patients or those requiring frequent monitoring. Advancements in low-dose CT protocols have helped mitigate this issue to some extent [53].

In contrast, MRI does not use ionizing radiation, making it particularly suitable for long-term monitoring and use in younger patients. This advantage becomes more pronounced when frequent imaging is necessary to track disease progression [54].

PET/CT involves radiation exposure from both the CT component and the radiotracer. PET/MRI reduces radiation exposure by eliminating the CT component but still involves radiotracer-related radiation [55].

Role of imaging in disease monitoring and treatment planning

Imaging plays a crucial role not only in the initial diagnosis of AO but also in monitoring disease progression and guiding treatment decisions. This section explores the various aspects of how imaging contributes to long-term patient management.

Follow-up Protocols

The chronic nature of AO necessitates regular imaging follow-ups. While there is no universally accepted protocol, imaging frequency is typically tailored to the individual patient's clinical status and disease severity [21].

For patients with stable symptoms, MRI is often recommended annually or biannually to assess for subtle changes in soft tissues and potential progression of calcification [5]. Low-dose CT may be considered every one to two years to evaluate the extent of calcification, especially if MRI findings are equivocal [3].

For patients with progressive symptoms or after interventional procedures, more frequent imaging, potentially every three to six months, may be necessary to closely monitor disease activity and treatment response [14]. A combination of MRI and CT might be employed to provide comprehensive information about both soft tissue changes and calcification progression [12].

Evaluating Disease Progression

Imaging plays a vital role in objectively assessing disease progression over time in AO. Serial CT or high-resolution MRI can quantify changes in the size, number, and distribution of calcifications [16]. MRI is particularly useful in monitoring for progressive cord compression or syrinx formation, which may necessitate surgical intervention [11]. Contrast-enhanced MRI can help assess ongoing inflammation, potentially guiding anti-inflammatory treatments [21]. Advanced MRI techniques like diffusion tensor imaging (DTI) can provide insights into the functional integrity of white matter tracts, offering a more comprehensive assessment of disease impact [26].

In cases with extensive ossification, CT can monitor for secondary bony changes or potential instability [1]. These various imaging modalities and techniques allow clinicians to track multiple aspects of the disease, including the extent of calcification, spinal cord compression, inflammatory activity, functional impact on neural structures, and associated bone changes. This comprehensive approach to monitoring disease progression enables more informed decision-making regarding treatment strategies and timing of interventions.

Guiding Interventional Procedures

Radiologically guided interventions for AO primarily aim to alleviate symptoms and improve neurological function. These include epidural steroid injections to reduce inflammation, selective nerve root blocks for targeted pain relief, and percutaneous lysis of adhesions to address the tethering of nerve roots [56]. Indications for these procedures typically involve intractable pain, progressive neurological deficits, or cases where surgery is contraindicated. However, these interventions have limitations, including temporary relief, the potential for infection, and the risk of further neural injury, particularly in areas of dense calcification [57]. The success of these procedures is often limited by the extent and location of ossification, highlighting the need for careful patient selection and meticulous technique guided by high-quality imaging.

High-resolution CT and MRI are essential for the precise localization of calcifications and assessment of their relationship to neural structures, crucial for surgical decision-making and approach [13]. Imaging guidance, often using fluoroscopy or CT, is vital for accurate needle placement in minimally invasive procedures such as epidural injections or selective nerve root blocks [56]. Advanced intraoperative imaging, including navigation systems based on preoperative imaging, can enhance the safety and efficacy of surgical interventions [57].

Post-procedure imaging plays a critical role in managing AO patients. Immediate post-procedure imaging can confirm the success of interventions and serve as a new baseline for future comparisons [9]. Follow-up imaging is crucial in evaluating the effectiveness of both conservative and surgical treatments, guiding decisions on whether to continue, modify, or change management strategies [26]. This comprehensive use of imaging throughout the treatment process enables clinicians to optimize patient care, minimize risks, and assess outcomes more effectively in the challenging management of AO.

Future directions

The field of radiological imaging for AO is rapidly evolving, with several promising research areas on the horizon. Promising research areas include the development of targeted molecular imaging techniques. Recent advancements in PET tracer development, such as those targeting neuroinflammation, could potentially be applied to AO, allowing for more specific visualization of disease activity [58].

The potential for AI and machine learning in image analysis is particularly exciting. Recent studies have demonstrated the successful application of deep learning algorithms in detecting and quantifying spinal pathologies [59]. These techniques could be adapted for AO, potentially improving early detection and automated assessment of disease progression [60].

Radiomics, an emerging field that extracts large amounts of quantitative features from medical images, shows promise for characterizing complex imaging patterns. This approach could provide new insights into the radiological manifestations of AO and aid in predicting disease outcomes [61].

Despite these advancements, significant gaps in current knowledge persist. The natural history of AO as visualized through longitudinal imaging studies remains poorly understood [62]. Additionally, the correlation between imaging findings and clinical symptoms needs further exploration to guide treatment decisions more effectively [3].

Areas for further study include standardization of imaging protocols for consistent evaluation across institutions, development of imaging biomarkers for early disease detection and progression monitoring, investigation of advanced MRI techniques, such as sodium imaging, for assessing calcification processes [63], and exploration of the potential role of hybrid imaging modalities like PET/MRI in comprehensive disease assessment [64]. These research directions aim to enhance the diagnostic accuracy and prognostic value of imaging in the management of the disease, ultimately improving patient outcomes.

## Conclusions

This narrative review has explored the evolving landscape of radiological approaches in the diagnosis and monitoring of AO. From the historical use of plain radiographs to the cutting-edge applications of high-field MRI, PET/CT, and emerging AI-assisted analysis, the field has witnessed significant advancements. These developments have enhanced our ability to detect, characterize, and monitor this rare and challenging condition. The comparative analysis of various imaging modalities highlights the importance of a multimodal approach, balancing sensitivity, specificity, cost-effectiveness, and radiation exposure considerations. As we look to the future, promising research areas such as molecular imaging, radiomics, and machine learning offer exciting possibilities for earlier diagnosis and more personalized patient management. However, significant gaps in our understanding persist, particularly regarding the correlation between imaging findings and clinical outcomes. Moving forward, standardization of imaging protocols, development of imaging biomarkers, and longitudinal studies will be crucial in addressing these knowledge gaps. Ultimately, the continued evolution of radiological approaches holds the potential to significantly improve the care and outcomes for patients with AO, underscoring the vital role of imaging in the management of this complex neurological condition.
